# SNHG22 promotes migration and invasion of trophoblasts via miR-128-3p/PCDH11X axis and activates PI3K/Akt signaling pathway

**DOI:** 10.1016/j.clinsp.2022.100055

**Published:** 2022-06-06

**Authors:** Xiaoying Wei, Yichong Yuan, Qiong Yang

**Affiliations:** a Department of Obstetrics, Maternal and Child Health Hospital of Hubei Province, Wuhan, Hubei, China; b Department of Gynaecology, Maternal and Child Health Hospital of Hubei Province, Wuhan, Hubei, China

**Keywords:** Preeclampsia, Trophoblast, SNHG22, miR-128-3p, PCDH11X, Migration, LncRNAs, Long non-coding RNAs, PE, Preeclampsia, SNHG22, Small nucleolar RNA host gene 22, PCDH11X, Protocadherin 11 X-Linked, EMT, Epithelial-mesenchymal transition, LASP1, LIM And SH3 Domain Protein 1, IGF1, Insulin Like Growth Factor 1, USAR, Uterine Spiral Artery Remodeling, UCA1, Urothelial Cancer Associated 1, JAK2, Janus Kinase 2, PUM1, Pumilio RNA Binding Family Member 1, HOTAIR, HOX Transcript Antisense RNA, AFAP1-AS1, AFAP1 Antisense RNA 1, DUSP5, Dual Specificity Phosphatase 5, CRIM1, Cysteine Rich Transmembrane BMP Regulator 1, PLT, Blood Platelet, AST, Aspartate Aminotransferase, ALT, Alanine Aminotransferase, BP, Blood Pressure

## Abstract

**Objectives:**

Long non-coding RNAs (LncRNAs) act as an indispensable role in the Preeclampsia (PE)-related trophoblast function, while its relationship with Small Nucleolar RNA Host Gene 22 (SNHG22) remains unknown. Hence, this study aimed to investigate the roles of lncRNA SNHG22 in the Preeclampsia (PE)-related trophoblasts function and the underlying mechanism.

**Method:**

Normal placentas and placentas from PE patients were collected to detect the expression of lncRNA SNHG22. Then, trophoblasts HTR-8/Svneo and JEG-3 were purchased, cultured, and treated to investigate the roles of lncRNA SNHG22 on cell migration and invasion as well as its underlying regulatory mechanism.

**Results:**

The SNHG22 was downregulated in PE patients, and it was found that SNHG22 overexpression could drive migration and invasion of trophoblasts, while SNHG22 depletion exerted a suppressive effect. Mechanistically, SNHG22 was validated to regulate microRNA-128-3p (miR-128-3p), and Protocadherin 11 X-Linked (PCDH11X) was identified as the target gene of miR-128-3p. Furthermore, it was found that SNHG22 acted as a promoter in the migration and invasion of trophoblast cells in a miR-128-3p/PCDH11X dependent manner, and SNHG22 silencing weakened the activation of PCDH11X-mediated PI3K/Akt signaling pathways through inhibiting miR-128-3p, thereby preventing migration and invasion of trophoblasts.

**Conclusion:**

SNHG22 acted as a driver in the migration and invasion of trophoblasts and may be considered a candidate for the amelioration of PE.

## Highlights

• SNHG22 in downregulated in placentas from PE patients.
• Overexpression of SNHG22 promotes the migration and invasion of trophoblasts.
• SNHG22 works as a sponger of microRNA-128-3p to increase PCDH11X expression in trophoblasts.
• SNHG22 modulates PCDH11X-activated PI3K/Akt signaling to drive migration and invasion of trophoblasts.

## Introduction

Preeclampsia (PE) is a unique progressive multi-systemic disease during pregnancy^1^ and is characterized by the emergence of hypertension and proteinuria after 20 weeks of pregnancy,^2^^,^^3^ which seriously threatens the health of both the mother and baby. The occurrence and development of PE are highly related to abnormal uterine-placental vascular structure,^4^ excessive activation of inflammatory immunity,^5^ vascular endothelial cell injury^6^ and genetic factors.^7^ In addition, it has been proved that trophoblast dysfunction significantly contributes to the induction of PE,^8^ and increasing evidence has highlighted that normal proliferation and migration of trophoblasts can prevent PE.^9^^,^^10^ Thus, it is necessary to identify indicators involved in the migration and invasion of trophoblasts for the prevention of PE.

Long non-coding RNA (LncRNA) is a type of molecule containing 200 nucleotides. LncRNA involves in a variety of biological processes, including chromatin modification, transcriptional activation, nuclear transport, and activation of disease-related genes at the epigenetic, transcriptional, or posttranscriptional levels.^11^^,^^12^ Recently, it has been indicated that LncRNA is related to PE progression. For instance, LncRNA SNHG16 has been unraveled to facilitate trophoblast proliferation and invasion by mediating miR-218-5p/LASP1 axis in preeclampsia,^13^ and Yin et al.^14^ demonstrated that lncRNA-ATB overexpression could drive migration and invasion of trophoblasts via upregulating the miR-651-3p-targeted YY1 pathway. In contrast, Chi et al.^15^ disclosed that the depleted LINC00473 induced the migration, invasion, and Epithelial-Mesenchymal Transition (EMT) process in preeclampsia through modulating miR-15a-5p/LITAF axis. LncRNA Small Nucleolar RNA host gene 22 (SNHG22) has been found to play a role in the migration and invasion of several carcinomas, such as gastric cancer, hepatocellular carcinoma, and breast cancer.^16^, ^17^, ^18^ However, the contribution of SNHG22 to the biological function of trophoblasts remains unclear.

MicroRNA belongs to small non-coding RNA molecules, which play a biological role via binding messenger mRNA.^19^ Accumulating studies have elaborated on the involvement of microRNAs in the onset and progression of PE. For example, according to Ma et al., miR-486-5p expressed in exosomes secreted by human placental microvascular endothelial cells could suppress proliferation and migration of trophoblasts through inhibiting IGF1,^20^ and Hayder et al.^21^ revealed that miR-210-3p could intensify USAR (uterine spiral artery remodeling)-related trophoblast dysfunction. In addition, Su et al. reported that miR-200 was highly expressed in PE patients and restricted EMT and invasion of trophoblasts in a ZEB1-dependent manner.^22^ Ding et al.^23^ recently reported that miR-128-3p is upregulated in placentas tissues and promotes the apoptosis of the trophoblasts, suggesting its contribution to PE. Whether miR-128-3p modulates trophoblast migration and invasion in PE has not been studied. In the present study, in order to ascertain whether SNHG22 mediates miR-128-3p via the ceRNA mechanism, the authors investigated the correlation between the two and investigated the roles of SNHG22/miR-128-3p/PCDH11X axis in the trophoblasts. These insights may be beneficial for the prevention and treatment of PE.

## Materials and methods

### Tissue collection

Placentas tissues from 50 women (25 patients diagnosed with preeclampsia and 25 age-matched normal pregnant women) who were hospitalized in the Maternal and Child Health Hospital of Hubei Province between January 2017 and November 2020 were enrolled in this study, and the study was performed following the guidelines of Declaration of Helsinki. The protocol was approved by the Ethics Committee of Maternal and Child Health Hospital of Hubei Province. Signed informed consent was obtained from all patients or their families. The diagnosis criteria of PE: systolic pressure ≥ 140 mmHg or diastolic pressure ≥ 90 mmHg after 20 weeks of gestation in a woman with previously normal blood pressure; proteinuria, excretion of ≥ 0.3g protein in a 24 h urine specimen.

The characteristics are provided in Table 1. Tissues were immediately collected from the basal plate when the placenta was delivered, and they were then cleaned three times with diethylpyrocarbonate-treated saline to wash out the blood and subsequently snap-frozen in liquid nitrogen. Then, the tissues were stored at -80 °C.

**Table 1:** Clinicopathological features of preeclampsia and normal pregnant women.

Characteristics	PE (*n* = 25)	Normal (*n* = 25)	*p*
Maternal age (y)	29.52 ± 4.00	28.44 ± 3.73	0.3286
Gestational age (wk)	34.76 ± 4.02	36.52 ± 3.69	0.1134
Weight (kg)	66.96 ± 9.38	62.74 ± 6.83	0.0751
Height (cm)	158.30 ± 4.60	158.16 ± 5.08	0.9213
Body Mass Index (kg/m^2^)	26.67 ± 3.12	25.09 ± 2.65	0.0603
Systolic BP (mm Hg)	134.92 ± 16.02	108.44 ± 9.47	<0.0001
Diastolic BP (mm Hg)	91.04 ± 12.01	67.6 ± 7.91	<0.0001
24h proteinuria quantification (g/24h)	2.65 ± 1.11	0.10 ± 0.03	<0.0001
Urine (mL/24 h)	499.12 ± 127.83	997.92 ± 201.70	<0.0001
PLT (10^9/L)	145.71 ± 47.61	218.50 ± 29.37	<0.0001
AST (U/L)	22.10 ± 8.49	16.51 ± 6.60	0.0124
ALT (U/L)	16.16 ± 9.73	12.02 ± 8.00	0.1066
Creatinine (μmoL/L)	77.36 ± 15.10	50.42 ± 9.97	<0.0001
Birth weight (kg)	2.41 ± 0.42	3.14 ± 0.26	<0.0001
Birth length (cm)	38.08 ± 4.93	46.24 ± 5.17	<0.0001
Apgar (1 min)	6.65 ± 0.82	7.96 ± 0.89	<0.0001

Note: 8 puerperae lack the data of 24h proteinuria quantification. Values are shown as mean ± SD. y, years; wk, weeks; PLT, Blood Platelet; AST, Aspartate Aminotransferase; ALT, Alanine Aminotransferase; BP, Blood Pressure.

### Cell culture and transfection

Two human trophoblast cell lines, HTR-8/Svneo and JEG-3, were both purchased from ATCC (American Type Culture Collection, USA) and were cultured in the medium with 10% FBS/DMEM under a humidified atmosphere with 5% CO_2_ at 37 °C. For cell transfection, SNHG22, short hairpin RNAs (shRNAs) targeting SNHG22 (sh-SNHG22), miR-128-3p-mimics, miR-128-3p-inhibitors, PCDH11X and Small interfering RNAs (siRNAs) against PCDH11X (si-PCDH11X) were designed by Gene Chem (Shanghai, China) (Supplementary Table 1), and those plasmids were transfected as per the guidance of lipofectamine (11668-019, Invitrogen, USA).

### Cells migration and invasion assays

For invasion analysis, the Transwell upper chamber was supplemented with 100 μL cellular suspension and serum-free medium, while the lower chamber was added with 500 μL medium and 10% FBS. The HTR-8/Svneo and JEG-3 cells were transfected and seeded (5 × 10^4^ cells) in the upper chambers of the transwell filters (8 μm; Corning Incorporated, Corning), which were pre-coated with Matrigel (BD Biosciences). The lower chambers were placed with a medium containing 10% FBS. After 24 h culture at 37 °C, the cotton swab was used to scrape cells in the upper chamber, and the cells were fixed and stained with paraformaldehyde and crystal violet. The cells were counted under a microscope, and cell counts under 5 different views were obtained.

A wound assay was performed to detect cell migration. The transfected HTR-8/Svneo and JEG-3 cells were seeded in a 6-well plate (1 × 10^6^°cells). A pipette tip after sterilization was used to scrape cell monolayers, an RPMI-1640 medium without FBS was added to each well, and cells were incubated for 48 h at 37 °C with 5% CO_2_. An inverted optical microscope was introduced to observe wound gaps based on the Zen Imaging software (Carl Zeiss, Oberkochen, Germany).

### Dual-luciferase reporter assay

To investigate the correlation between miR-128-3p with SNHG22 and PCDH11X, a dual-luciferase reporter assay was performed based on the manufacturer's instructions. In brief, miR-128-3p-mimics and miR-128-3p-inhibitors were co-transfected with HTR-8/Svneo and JEG-3 cells with SNHG22-WT or SNHG22-MUT/PCDH11X-MUT or PCDH11X-WT. Using Dual-Luciferase Reporter Assay System (Promega, Madison, United States), the luciferase activities of SNHG22 and PCDH11X were tested.

### Western blotting

To detect PI3K/Akt signaling pathway, the total proteins were extracted from HTR-8/Svneo cells using RIPA lysis buffer, and then SDS-PAGE of the samples was performed, and then the proteins were transferred onto a PVDF membrane (Millipore, MA) blocked by a blocking solution (Beyotime). Next, the membrane was incubated overnight with primary antibodies of PI3K/Akt signaling pathway-related proteins at 4°C, including p-PI3K (ab278545, 0.5 µg/mL), PI3K (ab40755, 1:2,000), Akt (ab8805, 1:500), p-Akt (T308) (ab38449, 1:500), p-Akt (S473) (ab81283, 1:5,000), and PCDH11X (LS-C167365, 1:1,000, abnova). After the membranes were washed, the HRP-conjugated secondary antibodies were added for further incubation. Enhanced Chemiluminescence Substrate (PerkinElmer, USA) was used for the visualization of protein bands.

### qRT-PCR

TRIzol reagent (Invitrogen, Carlsbad, USA) was used to extract RNA from PE placentas and trophoblast HTR-8/Svneo and JEG-3. Then PrimeScript Reverse Transcriptase (TaKaRa, Japan) was applied to the reverse transcription of extracted RNA, and the real-time quantification of RNA was achieved using LightCycler 480 (Roche, Basel, Switzerland). In this experiment, GAPDH and U6 were used as the internal reference, and the formula of 2^−ΔΔCT^ was used to calculate the relative expression of genes. The detailed primer sequences are shown in Supplementary Table 2.

### Bioinformatics and statistical analysis

The online tools TargetScan (http://www.targetscan.org/) and starBase (http://starbase.sysu.edu.cn/index.php) was used to analyze potential target genes of miR-128-3p. Relevant signaling pathways of PCDH11X were explored using Gene Set Enrichment Analysis (GSEA) (http://software.broadinstitute.org/gsea/index.jsp). For statistical analysis, all data were analyzed using GraphPad Prism (vision 5.01) software. Each experiment was conducted in triplicate. Quantitative data were expressed as mean ± Standard Deviation (SD). The differences between the two groups were assessed by Student's *t*-test, while ANOVA was employed to estimate the differences among multiple groups. Spearman's correlation analysis was used for the determination of correlation between two parameters; *p* < 0.05 indicated a significant difference.

## Results

### SNHG22 drives migration and invasion of trophoblasts

**Fig. 1:**
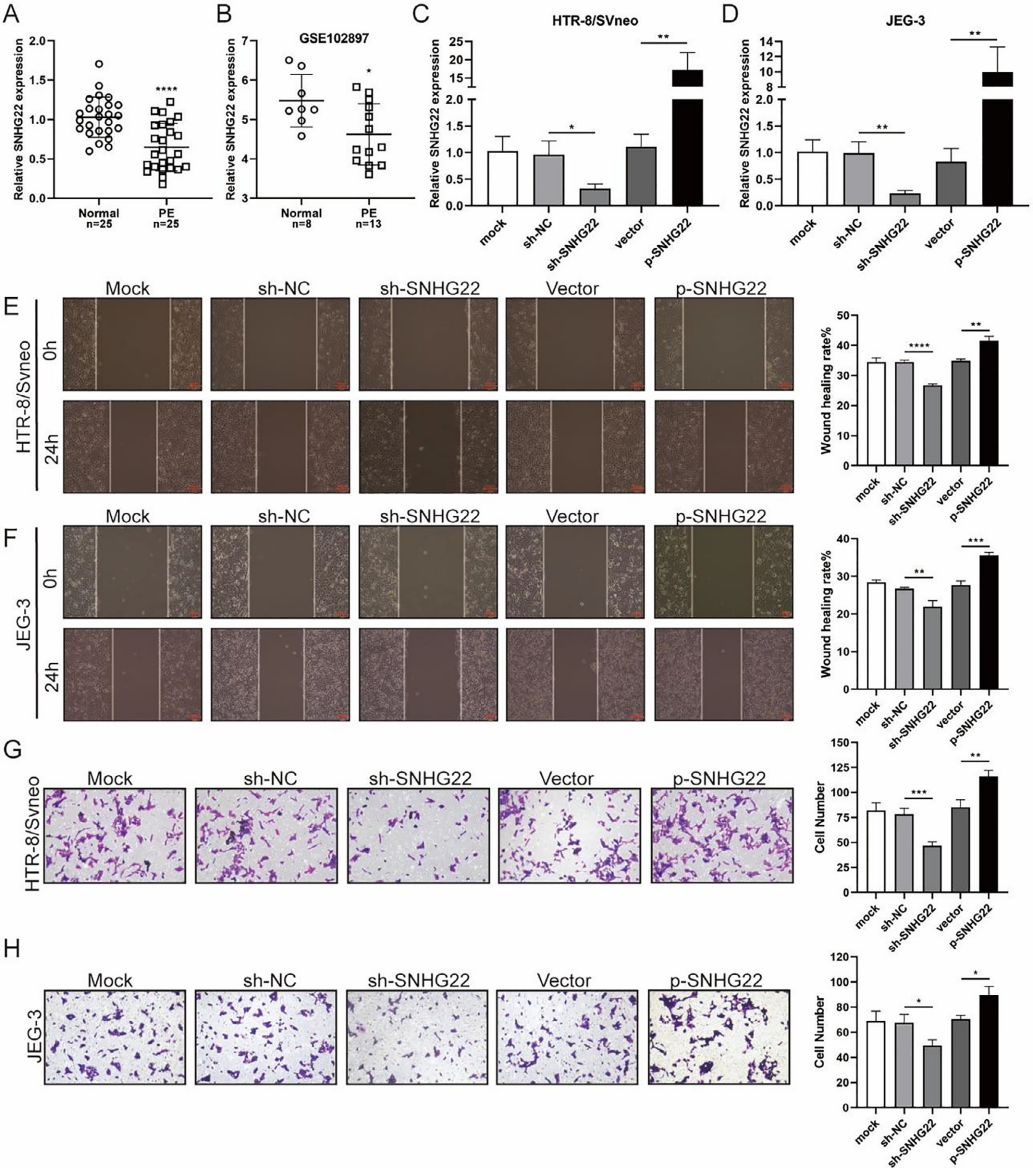
SNHG22 drives trophoblast migration and invasion. (A) SNHG22 expression in normal placenta (*n* = 25) and PE placenta (*n* = 25) tissues was revealed by qPCR test. (B) SNHG22 expression in normal placenta (*n* = 8) and PE placenta tissues (*n* = 13) was analyzed in a public database (GSE102897). (C and D). SNHG22 was overexpressed or silenced in trophoblasts, and the transfection efficacy was examined by a qPCR test. Wound healing (E and F) and Transwell (G and H) assays estimated the migration and invasion of trophoblasts after increasing or decreasing SNHG22 expression. Experiments were replicated three times. *p < 0.05, **p < 0.01, ***p < 0.001, ****p < 0.0001. Data are expressed as mean±SD.

To unravel the role of SNHG22 in PE, the authors compared the expression of SNHG22 in normal placentas and PE placentas tissues and public database (GSE102897), and it was found that the expression of SNHG22 was much lower in PE placentas (Fig. 1A and 1B). For further functional investigation, SNHG22-overexpressed or SNHG22-silenced HTR-8/Svneo and JEG-3 cells were constructed, and the transfection efficacy was validated by the results of q-PCR (Fig. 1C and D). Wound healing and Transwell assays showed that SNHG22 overexpression significantly improved the migration and invasion of HTR-8/Svneo and JEG-3 cells, while SNHG22 depletion showed the opposite effects (Fig. 1E‒1H). These findings implied that SNHG22 was downregulated in PE placentas and acted as a driver in the migration and invasion of trophoblasts.

### SNHG22 functions as a regulator of miR-128-3p in trophoblasts

**Fig. 2:**
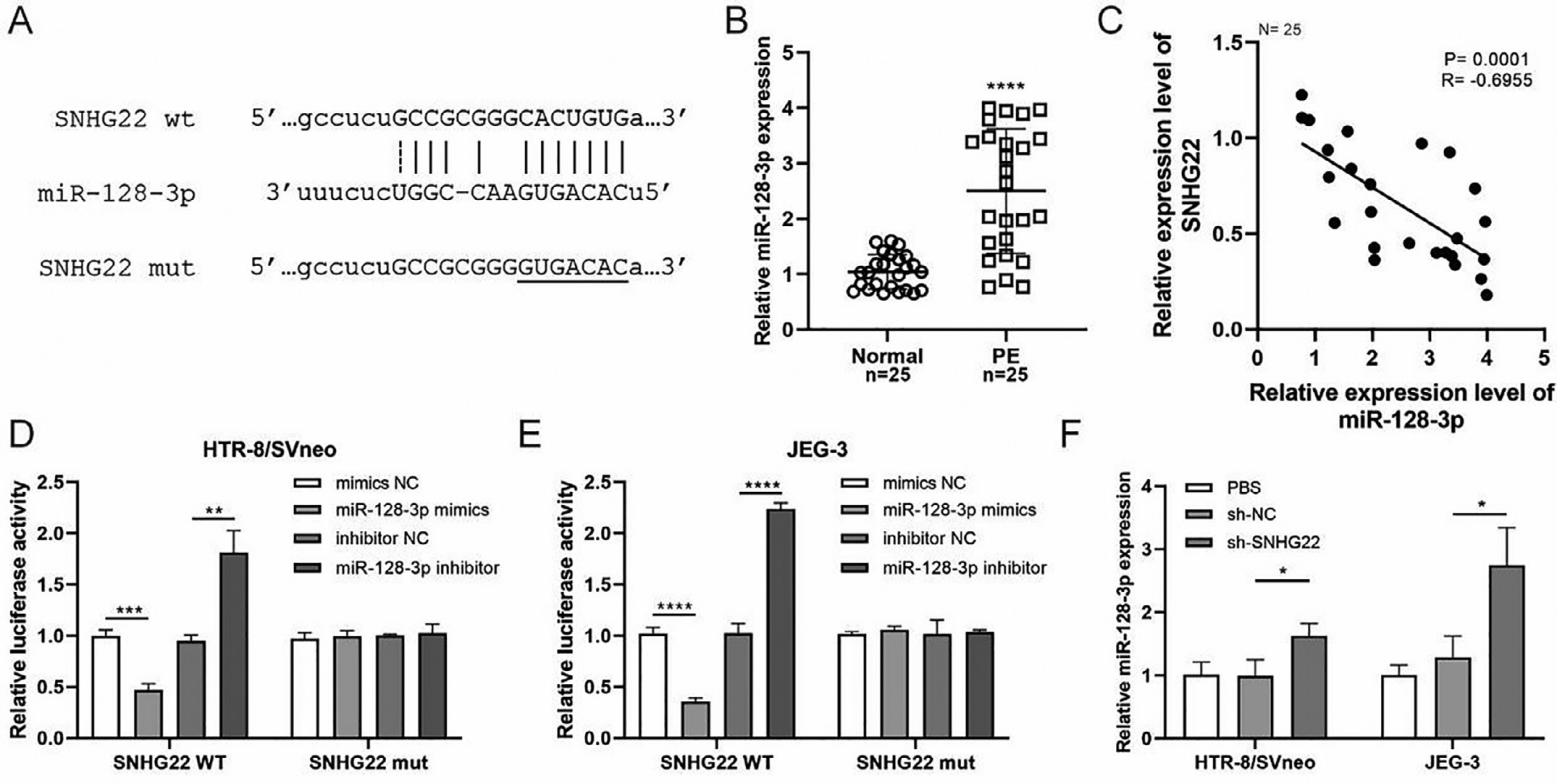
SNHG22 functions as a regulator of miR-128-3p in trophoblasts. Starbase database was used to predict the potential binding site between SNHG22 and miR-128-3p. (B) miR-128-3p expression in normal placenta (*n* = 25) and PE placenta (*n* = 25) tissues was determined by q-PCR. (C) Spearman's correlation analysis was applied to detect the correlation between the expression of miR-128-3p and SNHG22 in PE placenta tissues (*n* = 25). (D and E) HTR-8/Svneo and JEG-3 cells were co-transfected with miR-128-3p mimic or miR-128-3p inhibitor and SNHG22-WT or SNHG22-MUT. The luciferase activity was determined by a dual-luciferase reporter assay. (F) miR-128-3p expression in SNHG22-silenced HTR-8/Svneo and JEG-3 cells was determined by q-PCR. Experiments were replicated three times. *p < 0.05, **p < 0.01, ***p < 0.001, ****p < 0.0001. Data are expressed as mean ± SD.

To reveal the underlying mechanism, an online Starbase database was used to predict the target of SNHG22, and it was found that there was a binding site between SNHG22 and miR-128-3p (Fig. 2A). Notably, the expression of miR-128-3p was higher in PE placentas compared with that in normal placentas (*p* < 0.0001) (Fig. 2B), and Spearman's correlation analysis identified a negative correlation between the expression of miR-128-3p and SNHG22 (R = -0.6955, *p* = 0.0001) (Fig. 2C). Additionally, the dual-luciferase reporter assay showed that miR-128-3p mimics caused reduced luciferase activity of SNHG22-WT in both HTR-8/Svneo and JEG-3 cells, but miR-128-3p inhibitors resulted in the opposite effect (Fig. 2D and E). Furthermore, it was found that miR-128-3p was strikingly upregulated in SNHG22-depleted HTR-8/Svneo and JEG-3 cells (*p* < 0.05) (Fig. 2F). Collectively, these data indicated that SNHG22 could regulate miR-128-3p to inhibit its expression in trophoblasts.

### SNHG22 accelerates migration and invasion of trophoblasts via regulating miR-128-3p

**Fig. 3:**
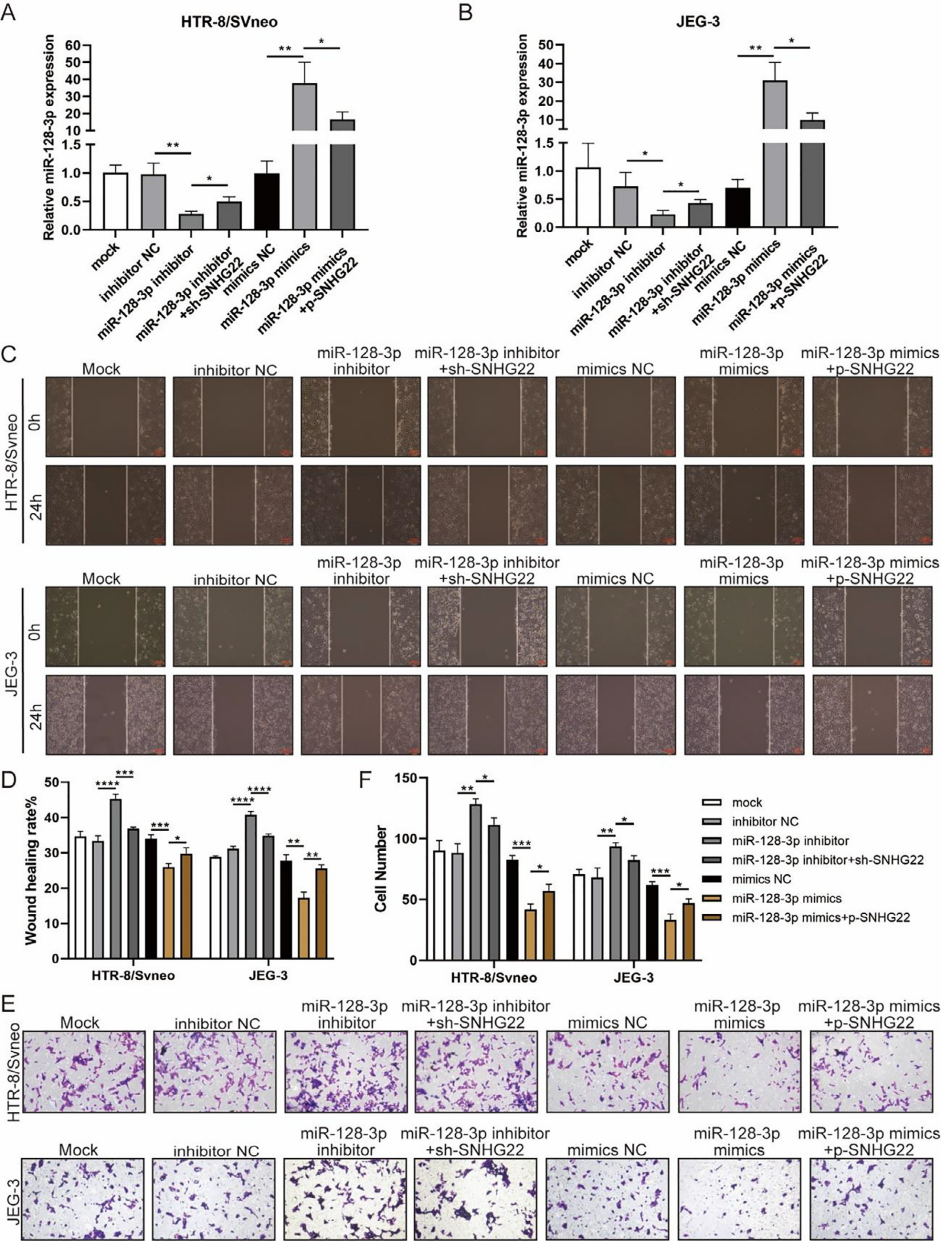
SNHG22 accelerates trophoblast migration and invasion via regulating miR-128-3p. HTR-8/Svneo and JEG-3 were transfected with inhibitor-NC, miR-128-3p inhibitor with or without sh-SNHG22, or mimics-NC, miR-128-3p mimics with or without pcDNA-SNHG22.After transfection for 24h, the cells were collected and performed further experiments. (A and B) miR-128-3p expression in HTR-8/Svneo and JEG-3 cells was examined by q-PCR. Wound healing (C and D) and Transwell assays (E and F) demonstrated the migration and invasion of HTR-8/Svneo and JEG-3 cells. Experiments were replicated three times. *p < 0.05, **p < 0.01, ***p < 0.001, ****p < 0.0001. Data are expressed as mean±SD.

To investigate whether SNHG22 acts as a promoter in the migration and invasion of trophoblasts in a miR-128-3p-dependent manner, rescue experiments were performed. MiR-128-3p inhibitors and sh-SNHG22 or miR-128-3p mimics and SNHG22 plasmids were co-transfected with HTR-8/Svneo and JEG-3 cells. It was found that levels of miR-128-3p were upregulated by SNHG22 depletion and downregulated by SNHG22 overexpression (Fig. 3A and B). Furthermore, wound healing and Transwell assays demonstrated that miR-128-3p depletion facilitated migration and invasion of HTR-8/Svneo and JEG-3 cells, which was blocked by SNHG22 silencing. In contrast, damages of migration and invasion induced by miR-128-3p overexpression could be recovered by SNHG22 overexpression (Fig. 3C‒F). Taken together, the authors confirmed the hypothesis that SNHG22 facilitated migration and invasion of trophoblasts through mediating miR-128-3p.

### miR-128-3p negatively modulates PCDH11X in trophoblasts

**Fig. 4:**
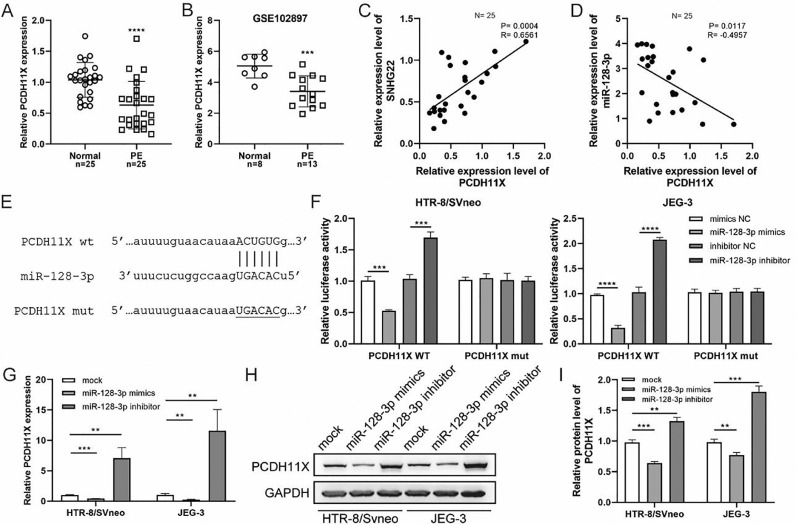
miR-128-3p negatively modulates PCDH11X in trophoblasts. (A) PCDH11X expression in normal placenta (*n* = 25) and PE placenta (*n* = 25) tissues was determined by q-PCR. (B) PCDH11X expression in normal placenta (*n* = 8) and PE placenta (*n* = 13) tissues was analyzed in a public database (GSE102897). (C and D) Spearman's correlation analysis was applied to detect the correlation between the expression of PCDH11X with SNHG22 (C) and miR-128-3p (D) in PE tissues (*n* = 25). (E) TargetScan database predicted a binding site between miR-128-3p and PCDH11X. (F) HTR-8/Svneo and JEG-3 cells were co-transfected with miR-128-3p mimic or miR-128-3p inhibitor and PCDH11X-WT or PCDH11X-MUT, the luciferase activity of was determined by dual-luciferase reporter assay. (G-I) HTR-8/Svneo and JEG-3 cells were transfected with miR-128-3p mimic or miR-128-3p inhibitor, q-PCR (G), and Western blotting (H and I) were performed to detect PCDH11X expression. Experiments were replicated three times. **p < 0.01, ***p < 0.001, **** *p* < 0.0001. Data are expressed as mean±SD.

Subsequently, q-PCR and GEO datasets showed the downregulation of PCDH11X in PE placentas (Fig. 4A and 4B), and a positive correlation between SNHG22 and PCDH11X was identified by Spearman's correlation analysis (R = 0.6561, *p* = 0.0004) (Fig. 4C), while a negative correlation between miR-128-3p expression and PCDH11X was found (R = -0.4957, p = 0.0117) (Fig. 4D). More importantly, the TargetScan database predicted a binding site between miR-128-3p and PCDH11X (Fig. 4E). Dual-luciferase reporter assay showed that miR-128-3p mimics resulted in reduced luciferase activity in the PCDH11X-WT group, but there were no changes in the PCDH11X-WT group (Fig. 4E). miR-128-3p inhibitors efficiently enhanced the luciferase activity in PCDH11X-WT (Fig. 4F). For further confirmation, miR-128-3p was overexpressed or silenced in HTR-8/Svneo and JEG-3 cells, and it was found that miR-128-3p overexpression suppressed PCDH11X mRNA (Fig. 4G) and proteins (Fig. 4H and 4I) levels of PCDH11X, whereas miR-128-3p depletion promoted the expression of PCDH11X mRNA and proteins. In summary, PCDH11X was confirmed as the target of miR-128-3p in trophoblasts.

### miR-128-3p hinders migration and invasion of trophoblasts by inhibiting PCDH11X

**Fig. 5:**
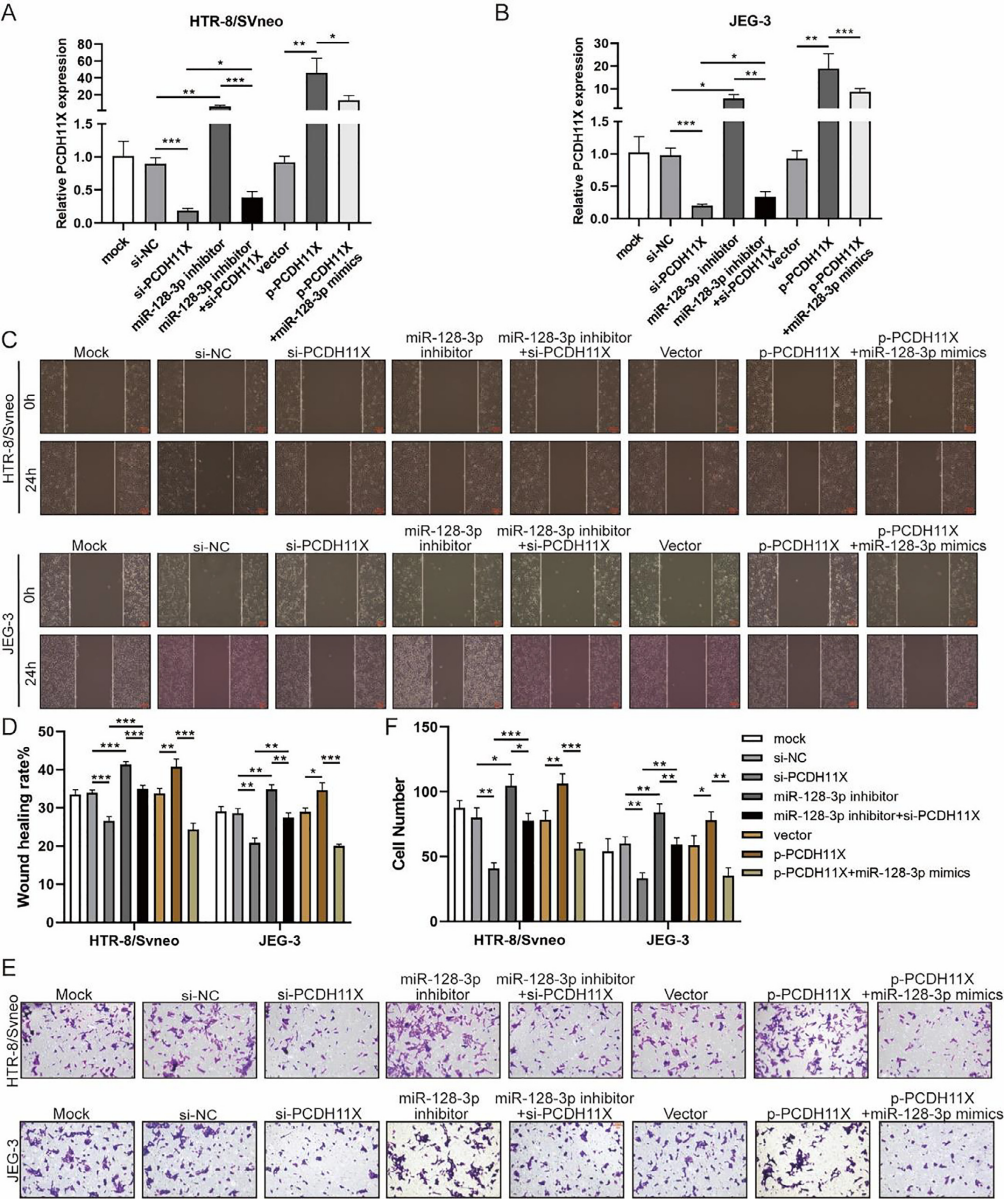
miR-128-3p hinders trophoblast migration and invasion by inhibiting PCDH11X. HTR-8/Svneo and JEG-3 were transfected with si-NC, si-PCDH11X with or without miR-128-3p inhibitor, or p-NC, p-PCDH11X with or without miR-128-3p mimics. After transfection for 24h, the cells were collected and performed the further experiments. (A and B) miR-128-3p expression in HTR-8/Svneo (A) and JEG-3 (B) cells were examined by q-PCR. Wound healing (C and D) and Transwell assays (E and F) assessed the migration and invasion of HTR-8/Svneo and JEG-3 cells. Experiments were replicated three times. *p < 0.05, **p < 0.01, ***p < 0.001. Data are expressed as mean ± SD.

To determine the role of PCDH11X in trophoblasts and whether miR-128-3p exerts its regulatory effect via PCDH11X, miR-128-3p and PCDH11X were co-depleted or co-overexpressed in HTR-8/Svneo and JEG-3 cells. Obviously, upregulation of PCDH11X induced by PCDH11X-depletion could be rescued by miR-128-3p inhibition, and overexpressed miR-128-3p blocked the upregulation of PCDH11X (Fig. 5A and 5B). In addition, it was found that PCDH11X depletion restricted migration and invasion of HTR-8/Svneo and JEG-3 cells, which could be retained by miR-128-3p inhibition. In contrast, upregulated PCDH11X acted as an enhancer on the cell migration and invasion, but it was blocked by co-transfection of miR-128-3p mimics (Fig. 5C‒F). These data indicated miR-128-3p exerted a repressive effect on trophoblast development in a PCDH11X-dependent manner.

### SNHG22 modulates PCDH11X-activated PI3K/Akt signaling to drive migration and invasion of trophoblasts

**Fig. 6:**
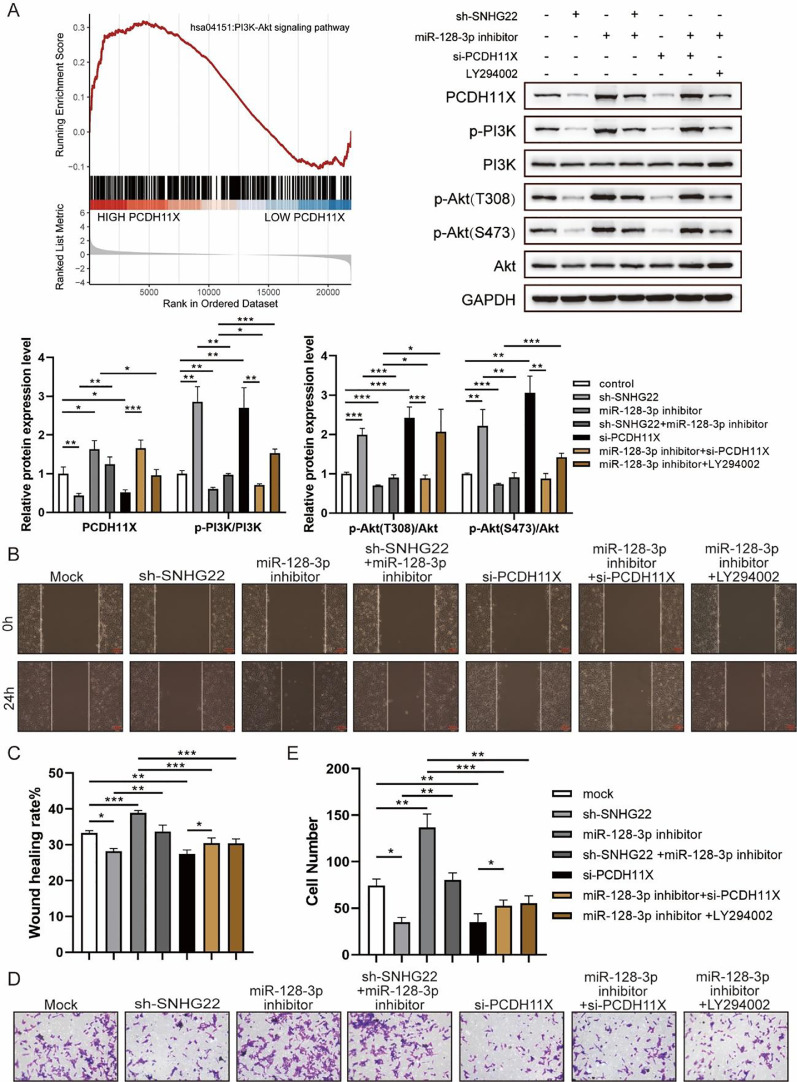
SNHG22 modulates PCDH11X-activated PI3K/Akt signaling to drive trophoblast migration and invasion. (A) GSEA and Western blotting analyzed the correlation of PCDH11X with PI3K/Akt signaling. HTR-8/Svneo and JEG-3 were transfected with si-NC, si-PCDH11X with or without miR-128-3p inhibitor. Wound healing (B and C) and Transwell assays (D and E) assessed the migration and invasion of HTR-8/Svneo cells. Experiments were replicated three times. *p < 0.05, **p < 0.01, ***p < 0.001. Data are expressed as mean ± SD.

For further exploration, the authors introduced Gene Set Enrichment Analysis (GSEA), and it was found that PCDH11X was highly correlated with PI3K/Akt signaling (Fig. 6A). For validation, SNHG22, miR-128-3p, and PCDH11X were downregulated in HTR-8/Svneo cells, and it was found that the phosphorylation level of PI3K/Akt signaling was enhanced by miR-128-3p inhibition but blocked by SNHG22 or PCDH11X depletion. In addition, the rescue experiments demonstrated that SNHG22 promoted PCDH11X-mediated PI3K/Akt signaling activation via regulating miR-128-3p (Fig. 6A). Furthermore, HTR-8/Svneo cells were treated with PI3K/Akt signaling inhibitors LY294002 and miR-128-3p inhibitors, and it was found that LY294002 could partly block enhanced migration and invasion of trophoblasts induced by miR-128-3p depletion (Fig. 6B‒E). Overall, the results implied that SNHG22 activates PI3K/Akt signaling pathway to accelerate migration and invasion of trophoblasts via mediating miR-128-3p/PCDH11X.

## Discussion

PE is a multisystem pregnancy disorder, and trophoblasts have been regarded as a source of nutrients for embryonic development and could promote the formation and development of placentas, which plays an indispensable role in exchanging the nutrients, waste, and gas between the maternal and fetal systems.^24^ In addition, Chambers et al. demonstrated that the biological behaviors of trophoblast cells, such as proliferation, migration, and invasion, are highly associated with the normal development of placentas.^25^ As the authors have unraveled in Table 1, women with PE were characterized with higher levels of proteinuria AST, ALT, creatinine, and lower Apgar score of newborn infants, suggesting the severity of PE on puerpera and newborns. So, it calls for the identification of indicators involved in PE.

Up to date, an increased number of LncRNAs have been identified as mediators of trophoblast function in PE. For example, Liu et al.^26^ indicated that lncRNA UCA1 suppressed JAK2 to prevent proliferation and invasion of trophoblasts, and Zhang et al.^27^ found that PUM1 was highly expressed in preeclampsia and could damage the capacities of trophoblast invasion through inhibiting lncRNA HOTAIR. In addition, lncRNA AFAP1-AS1 silencing epigenetically caused the upregulation of DUSP5, thereby restricting trophoblast proliferation and migration in PE.^28^ Similarly, in the present study, it was found that SNHG22 was downregulated in PE placentas, and overexpression of SNHG22 promoted migration and invasion of HTR-8/Svneo and JEG-3 cells, while they were inhibited by SNHG22 silencing, which confirmed the contribution of SNHG22 to biological behaviors of trophoblasts.

Accumulating evidence has revealed that lncRNAs exert their function through sponging miRNAs.^29^^,^^30^ Herein, the authors identified miR-128-3p as the target of SNHG22. More importantly, based on the results of rescue experiments, miR-128-3p inhibition enhanced trophoblast migration and invasion, which was blocked by SNHG22 silencing. In contrast, overexpressed SNHG22 recovered loss of migration and invasion of trophoblasts induced by miR-128-3p overexpression.

Furthermore, it was observed that miR-128-3p inhibited PCDH11X via binding to its 3′-UTR, and PCDH11X was positively correlated with SNHG22 and negatively associated with miR-128-3p. Moreover, it was unraveled that upregulated miR-128-3p repressed trophoblast migration and invasion through inhibiting PCDH11X expression. Consistent with the results, early studies have shown the crucial roles of miRNAs in the biological function of trophoblasts. For instance, miR-373-3p has been proved to suppress migration and invasion of trophoblasts by targeting CD44 and Radixin,^31^ and Hayder et al.^21^ highlighted that miR-210-3p upregulation could damage USAR-related biological functions of extravillous trophoblasts. In contrast, Jiang et al.^32^ reported that miR-335 facilitated the proliferation of the placental trophoblasts and suppressed their apoptosis by inhibiting CRIM1 in preeclamptic rats. Interestingly, according to previous evidence, PI3K/Akt signaling pathway significantly contributed to the development of trophoblasts.^9^^,^^33^ Herein, it was demonstrated that SNHG22 could activate PI3K/Akt signaling pathway to accelerate trophoblast migration and invasion via mediating miR-128-3p/PCDH11X. Although the authors displayed sufficient rationales to support the critical role of SNHG22 in promoting biological behaviors of trophoblasts, the authors’ findings remain to be further investigated by *in vivo* studies.

There are some limitations of the current study. In this study, it was found that SNHG22 promotes migration and invasion of trophoblasts. However, changes in SNHG22 in developed preeclampsia may just reflect the compensatory mechanisms the placenta undergoes in this condition but are not causative. Future studies are still needed to reveal the pathogenic cause of PE.

In conclusion, SNHG22 was confirmed as a driver in trophoblast migration and invasion, and it was revealed that SNHG22 exerted its function through the miR-128-3p/PCDH11X/PI3K/Akt pathway, which may provide new insight into amelioration of PE.

## CRediT authorship contribution statement

**Xiaoying Wei:** Conceptualization, Visualization, Investigation, Formal analysis, Writing – original draft, Writing – review & editing. **Yichong Yuan:** Conceptualization, Visualization, Investigation, Formal analysis, Writing – original draft, Writing – review & editing. **Qiong Yang:** Conceptualization, Visualization, Investigation, Formal analysis, Writing – original draft, Writing – review & editing.

## Conflicts of interest

The authors declare no conflicts of interest.

## Appendix Supplementary materials

Image, application 1
