# Supplementary material for: SNHG22 promotes migration and invasion of trophoblasts via miR-128-3p/PCDH11X axis and activates PI3K/Akt signaling pathway
Source: Clinics (Sao Paulo). 2022 Jun 6;77:100055. doi: 10.1016/j.clinsp.2022.100055 (PMC9188964; doi:10.1016/j.clinsp.2022.100055)
Supplement: Supplementary file 1 [file mmc1.docx]

**CLINICS-2021-3535_Supplementary Material**

**Supplementary Table 1. Sequences of siRNA Against Specific Targets.**

| sh-SNHG22 | 5’-3' | GCTCCTCTGTACAATATTA |
| --- | --- | --- |
| si-PCDH11X | 5’-3’ | GCCACTGATTCGAATTGAAGAGG |

**Supplementary Table 2. Sequences of PCR primers used in this study.**

| SNHG22 | Forward(5’-3’) | CTAAGAGTGGCCTCTGCGTG |
| --- | --- | --- |
|  | Reverse(5’-3’) | CAAGGCACCTAACAGGGGAG |
| miR-128-3p | Forward(5’-3’) | CTCAACTGGTGTCGTGGAGTCGGCAATTCAGTTGAGAAAGAGAC |
|  | Reverse(5’-3’) | ACACTCCAGCTGGGTCACAGTGAACCGGT |
| PCDH11X | Forward(5’-3’) | AACACCTTTGTGGCCTGTGA |
|  | Reverse(5’-3’) | GCCTCTTCCACAGTTGGTTG |
| GAPDH | Forward(5’-3’) | CAGGAGGCATTGCTGATGAT |
|  | Reverse(5’-3’) | GAAGGCTGGGGCTCATTT |
| U6 | Forward(5’-3’) | CTCGCTTCGGCAGCACACTCGCTTCGGCAGCACA |
|  | Reverse(5’-3’) | AACGCTTCACGAATTTGCGT |
